# Spatial image gradient estimation from the diffusion MRI profile

**DOI:** 10.1101/2025.06.06.658348

**Published:** 2025-09-15

**Authors:** Iman Aganj, Thorsten Feiweier, John E. Kirsch, Bruce R. Fischl, Andre J. van der Kouwe

**Affiliations:** 1. Athinoula A. Martinos Center for Biomedical Imaging, Radiology Department, Massachusetts General Hospital, Harvard Medical School, Boston, MA, USA; 2. Research & Clinical Translation, Magnetic Resonance, Siemens Healthineers AG, Erlangen, Germany

**Keywords:** Diffusion MRI (dMRI), spatial gradient, relaxation time, stimulated echo (STE)

## Abstract

**Purpose:**

In the course of diffusion, water molecules experience varying values for the relaxation-time properties of the underlying tissue. This factor, which has rarely been accounted for in diffusion MRI (dMRI) modeling, is modeled in this work, allowing for the estimation of the gradient of the relaxation-time properties from the dMRI signal.

**Methods:**

With the aim of mining the dMRI signal for information about the spatial variations in the tissue relaxation-time properties, a new mathematical relationship between the diffusion signal and the spatial gradient of the image is derived, which enables the estimation of the latter from the former. The hypothesis was validated on human brain dMRI images from three datasets: the public Human Connectome Project Young Adults database, 10 healthy volunteers and 1 *ex vivo* sample scanned in-house with stimulated-echo diffusion encoding and a long diffusion time of 1 second (which will be made publicly available), and 3 subjects from the public Multi-TE database. The effects of the confounding factor of “fiber continuity” were furthermore measured.

**Results:**

The image spatial gradient estimated from the diffusion signal was compared to the gold-standard spatial gradient approximated through the finite difference. The former gradient was significantly related to the latter in all datasets (i.e., with a difference significantly smaller than chance), with an effect distinct from fiber continuity.

**Conclusion:**

The results support the hypothesized relationship between within-voxel dMRI signal and image gradient, with an effect that was not explainable by the confounding factor of fiber continuity.

## 1. Introduction

As a noninvasive imaging modality, diffusion-weighted magnetic resonance imaging (dMRI) provides a wealth of information that has been proven valuable in revealing the microarchitecture of the neural tissue (1,2). Many imaging biomarkers for neurodegenerative diseases have been derived from dMRI in the past few decades (3), notably through the *in vivo* quantification of structural brain connectivity (4,5). Developing mathematical models consistent with dMRI physics and suitable to analyze dMRI data, which are inherently high-dimensional, has been necessary to extract the desired information about the tissue from the images (6–8).

Per the standard MRI model described by the Bloch equations, the MRI signal is proportional to the mean proton density (PD) inside a voxel, weighted according to relaxation times (RTs) of the tissue (9). In dMRI, where additional diffusion-encoding gradients are imposed, the signal is further attenuated with the displacement of water molecules in the direction of these gradients. Such a change in the measured signal enables inference of the diffusivity of water along different orientations, and hence the estimation of tissue properties such as fiber orientations (10,11). While the tissue RTs and diffusivity are distinct properties, they have been shown to be related to each other both theoretically (12–15) and empirically (16–20). The relationship between the two has been particularly explored in the context of diffusion-relaxation correlation (21). A number of studies have also examined how the dMRI signal is confounded by the presence of local magnetic field gradients, especially by the effects of susceptibility, at the mesoscopic scale (22–26).

Given the finite resolution of MRI, i.e. the measured signal being the average of continuous values inside a voxel, inferring within-voxel variations of an estimated quantity is often nontrivial at best. It is thus common to make approximations in MRI signal modeling by discounting such within-voxel variations. An example of such a scenario, which is the focus of this work, is in dMRI signal modeling. Although the interplay between the diffusion of spin-carrying molecules and the measured RTs has been thoroughly discussed (12–15), the effects of the spatial variations of the latter have rarely been accounted for in dMRI modeling. An exception is the work by Novikov *et al* (15), where the effects of the intrinsic variations of the transverse relaxation rate on the measured dMRI signal have been rigorously examined, and a general integral relationship between the apparent diffusion coefficient (ADC) and the Fourier transform of the autocorrelation function of the transverse relaxation rate is provided. Otherwise, standard dMRI models assume the RT properties of the tissue (resulting in T_1_ and T_2_ weighting) to be constant inside the voxel, an assumption that disregards the (small) contribution of the within-voxel variations of these properties to the dMRI signal.

In this work, we formulate and test a hypothesis based on the premise that, in the course of diffusion, water molecules experience varying values for the RT properties of the underlying tissue. We hypothesize that the spatial variation in tissue RTs (which is related to image gradient) affects the dMRI signal, thereby creating a relationship between the diffusion profile measured by dMRI and the spatial gradient of the image intensity. We derive this mathematical relationship and propose an approach to estimating the spatial gradient of the image from the dMRI signal (independently at each voxel). We validate our model via experiments on human brain dMRI data, specifically public images from the Human Connectome Project (HCP) Young Adult database (27), stimulated-echo (STE) (28,29) images that we acquired at our Center (and will soon make publicly available; see Section 6), and the public Multi-TE (MTE) dataset (30). The STE data were acquired with a very long diffusion time of one second, which our model predicts should increase the hypothesized effect size.

We have previously presented a preliminary abstract of this work (31). In the following, we will describe our model and methods (Section 2), present experimental results (Section 3), discuss them (Section 4), and provide our code and data (Section 6).

## 2. Methods

### 2.1. Signal Modeling

The Stejskal-Tanner pulsed gradient spin-echo sequence (10) applies two gradient pulses $\vec{G}$ of duration δ, separated in time by Δ. Molecules located at ${\vec{x}}_{0}$ during the first pulse and ending up at $\vec{x}={\vec{x}}_{0}+\vec{u}$ at the second pulse presumably contribute the following to the dMRI signal $S^{v}(\vec{q})$ at voxel *v*, where $\vec{q}:=\gamma \delta \vec{G}$ is the *q*-vector (or 2π times it with an alternative definition) with γ the gyromagnetic ratio (32):

[1] $$S^{v}(\vec{q})=\int_{v}w\left({\vec{x}}_{0}\right)\rho \left({\vec{x}}_{0}\right)\text{Pr}\left(\vec{x}|{\vec{x}}_{0}\right)e^{-i\vec{q}\cdot \left(\vec{x}-{\vec{x}}_{0}\right)}\text{d}{\vec{x}}_{0}\text{d}\vec{x}\;\;=\int_{v}w\left({\vec{x}}_{0}\right)\rho \left({\vec{x}}_{0}\right)\text{d}{\vec{x}}_{0}\int \text{Pr}\left(\vec{u}|{\vec{x}}_{0}\right)e^{-i\vec{q}\cdot \vec{u}}\text{d}\vec{u}\;\;=S_{0}^{v}{\hat{P}}^{v}(\vec{q}),$$

where ρ is PD, and $w\left({\vec{x}}_{0}\right):=\left[1-exp\left(-TR/T_{1}\left({\vec{x}}_{0}\right)\right)\right]exp\left(-TE/T_{2}\left({\vec{x}}_{0}\right)\right)$ is the RT weighting for the repetition time *TR*, echo time *TE*, and the tissue longitudinal and transverse RTs, $T_{1}\left({\vec{x}}_{0}\right)$ and $T_{2}\left({\vec{x}}_{0}\right)$, respectively. $S_{0}^{v}:=\langle w\rangle_{\rho }^{v}:=\int_{v}w\left({\vec{x}}_{0}\right)\rho \left({\vec{x}}_{0}\right)d{\vec{x}}_{0}$ is the baseline non-diffusion-weighted (i.e., b=0) image, where $\langle \cdot \rangle_{\rho }^{v}$ denotes ρ-weighted sum inside *v*, i.e. the voxel. ${\hat{P}}^{v}:=ℱ\left\{P^{v}\right\}$ is the Fourier transform of $P^{v}(\vec{u}):=Pr(\vec{u})≅Pr\left(\vec{u}|{\vec{x}}_{0}\right)$, which is the probability of diffusion with the amount $\vec{u}$ (a.k.a. ensemble average propagator) during the effective diffusion time τ:=Δ−δ/3, and is presumed (32) independent of ${\vec{x}}_{0}\in v$ within the voxel (although it could be considered dependent on the tissue type in multicompartment models).

The RT properties of tissue have been assumed to be constant along the trajectory of the diffusing water, thereby simplifying the above model. Given that the spatial distribution of molecules diffusing from ${\vec{x}}_{0}$ to $\vec{x}$ is their *initial* density, the integrals in Eq. [1] are weighted by $\rho \left({\vec{x}}_{0}\right)$. However, *w* is expected to vary in the tissue continuum along the molecule’s trajectory, which would affect the resulting signal attenuation. To account for the variations of *w*, the integral needs to be weighted by an *effective* value of *w* experienced by the molecules going from ${\vec{x}}_{0}$ to $\vec{x}$, rather than by its initial value at ${\vec{x}}_{0}$. Thus, weighting the above integral by $w\left({\vec{x}}_{0}\right)$, as done in the state-of-the-art dMRI models, neglects how the within-voxel variation of *w* affects the dMRI signal. Such effects could in fact be exploited to further learn about the tissue microstructure.

We propose to use an effective value for the RT weighting, w, to account for its change during a molecule’s diffusion. For particles going from ${\vec{x}}_{0}$ to $\vec{x}$, instead of weighting the integral in Eq. [1] by the initial value $w\left({\vec{x}}_{0}\right)$, we will use the *midpoint* value,

[2] w(12(x⇀0+x⇀))=w(x⇀0+12u⇀)≅w(x⇀0)+12∇xw(x⇀0)·u⇀,

where $\nabla_{x}$ is the spatial gradient. This leads to:

[3] Sv(q⇀)=∫vw(x⇀0)ρ(x⇀0)dx⇀0∫Pv(u⇀)e−iq⇀·u⇀du⇀+12∫v∇xw(x⇀0)ρ(x⇀0)dx⇀0·∫u⇀Pv(u⇀)e−iq⇀·u⇀du⇀=〈w〉ρvℱ{Pv(u⇀)}+12〈∇xw〉ρv·ℱ{u⇀Pv(u⇀)}=S0vP^v(q⇀)+12i〈∇xw〉ρv·∇qP^v(q⇀),

where we used the relationship $ℱ\left\{\vec{u}P^{v}(\vec{u})\right\}=i\nabla_{q}{\hat{P}}^{v}(\vec{q})$, with $\nabla_{q}$ the gradient with respect to $\vec{q}$. Note that our linear approximation of *w* in Eq. [2] is local only within the molecule’s trajectory and does not extend to the entire voxel.

Provided the measurements of the diffusion signal $S^{v}(\vec{q})$ for many *q*-vectors, as is common in dMRI, Eq. [3] allows the estimation of ${\left\langle \nabla_{x}w\right\rangle }_{\rho }^{v}$, i.e. the mean $\nabla_{x}w$ (weighted by PD) within the voxel, which can reveal potentially new information about the tissue microarchitecture. By contributing to the imaginary part of the signal, $\nabla_{x}w$ affects both the magnitude and the phase of the signal. It has been shown that the background phase varies strongly from one diffusion image to another in the scan (due to physiological factors) (33,34), which would render the estimation of the contribution of ${\left\langle \nabla_{x}w\right\rangle }_{\rho }^{v}$ to the phase image impractical. The *magnitude* of the signal, which, in contrast, remains largely consistent from shot to shot, would be:

[4] |Sv(q⇀)|=S0vP^v(q⇀)1+(L⇀v·12∇qlogP^v(q⇀))2,

where $\vec{L^{v}}:={\left\langle \nabla_{x}w\right\rangle }_{\rho }^{v}/S_{0}^{v}$, and we made the standard assumption that diffusion is symmetric (thus ${\overset{ˆ}{P}}^{v}(\vec{q})$ is real), as well as used $\nabla_{q}{\hat{P}}^{v}(\vec{q})/{\hat{P}}^{v}(\vec{q})=\nabla_{q}log{\hat{P}}^{v}(\vec{q})$. In the simple case of Gaussian diffusion, the diffusion tensor imaging (DTI) model (11) predicts ${\overset{ˆ}{P}}^{v}(\vec{q})=exp(-\tau {\vec{q}}^{T}D^{v}\vec{q})$, where $D^{v}$ is the symmetric diffusion tensor at voxel *v*, resulting in the factor $1/2\nabla_{q}log{\overset{ˆ}{P}}^{v}(\vec{q})=-\tau D^{v}\vec{q}$ being a linear function of $\vec{q}$. The DTI approximation, therefore, leads to:

[5] $$\left|S^{v}(\vec{q})\right|≅S_{0}^{v}e^{-\tau {\vec{q}}^{T}D^{v}\vec{q}}\sqrt{1+{\left(\tau {\vec{L}}^{v^{T}}D^{v}\vec{q}\right)}^{2}}.$$

With this model, measurements of $\left|S^{v}(\vec{q})\right|$ for at least 9 values of $\vec{q}$ in different orientations (in addition to an $S_{0}^{v}$ image) would be required to estimate the 6 parameters of $D^{v}$ and the 3 elements of the vector ${\vec{L}}^{v}$ (and subsequently ${\left\langle \nabla_{x}w\right\rangle }_{\rho }^{v}$). Note that, since ${\vec{L}}^{v}$ is raised to the power of 2 in Eq. [5], its sign cannot be directly recovered from $\left|S^{v}(\vec{q})\right|$ measurements, meaning that its magnitude and *orientation* (rather than direction) can be estimated. Including the fourth-order terms in $log{\hat{P}}^{v}(\vec{q})$ would extend the model to take advantage of diffusional kurtosis imaging (35), which is more accurate than DTI when the diffusion deviates from Gaussian (but requires 21 + 3 = 24 unknowns to be estimated from at least two q-shells).

### 2.2. Implementation and Assessment

To estimate $D^{v}$ and ${\vec{L}}^{v}$ at each voxel *v*, we fit the values of the diffusion signal for all available $\vec{q}$ to Eq. [5] by minimizing the residual sum of squares via the pattern search algorithm (36). We facilitate this nonconvex optimization by providing an initial solution close to the global minimum, namely by initializing $D^{v}$ with standard DTI reconstruction (11) and $\vec{L^{v}}$ as a 3×1 vector of all zeros. However, since the objective function is symmetric with respect to ${\vec{L}}^{v}$ about ${\vec{L}}^{v}=0$, if the initial $D^{v}$ were also the conditional minimizer of the function (with ${\vec{L}}^{v}$ constrained to be zero, i.e. standard DTI), then the aforementioned initialization would be counterproductive, as such an initial point would already be a local optimum with an objective-function gradient of zero with respect to both $D^{v}$ and ${\vec{L}}^{v}$. In our experiments, however, we did not face this issue thanks to the fact that the DTI reconstruction was performed with an existing tool (with a slightly different implementation), resulting in an initial $D^{v}$ that was close but not identical to the conditional minimizer of our objective function.

We assess the orientational accuracy of the estimated ${\vec{L}}^{v}$ by comparing it to its discrete counterpart (used as the gold standard) computed via finite difference, testing the hypothesis that the two orientations are more aligned than expected by chance. Where the PD image, $\rho^{v}=\langle 1\rangle_{\rho }^{v}$, is available, the discrete counterpart of ${\left\langle \nabla_{x}w\right\rangle }_{\rho }^{v}$ would be $\langle 1\rangle_{\rho }^{v}\partial_{fd}(\langle w\rangle_{\rho }^{v}/\langle 1\rangle_{\rho }^{v})=\rho^{v}\partial_{fd}\left(S_{0}^{v}/\rho^{v}\right)$, with $\partial_{fd}$ denoting the finite-difference gradient operator. The discrete counterpart of $\vec{L^{v}}$ can therefore be computed as the following vector:

[6] $$\partial_{\text{fd}}\left(\frac{S_{0}^{v}}{\rho^{v}}\right)/\left(\frac{S_{0}^{v}}{\rho^{v}}\right)≅\partial_{\text{fd}}\text{log}\left(\frac{S_{0}^{v}}{\rho^{v}}\right)\;\;=\partial_{\text{fd}}\text{log}\left(S_{0}^{v}\right)-\partial_{\text{fd}}\text{log}\left(\rho^{v}\right).$$

However, since the PD image is often unavailable in dMRI datasets, we approximate the above discrete counterpart with its first term, $\partial_{fd}log\left(S_{0}^{v}\right)$. Given that the *S*_0_ and PD image intensities both correlate with the same underlying tissue, their gradient *orientations* are expected to align with each other, favoring our approximation.

We measure the acute angle $\left(0\leq \theta \leq 90^{\circ }\right)$ between the orientations of the estimated $\vec{L^{v}}$ and its discrete counterpart, which should be small if the two are similarly oriented. The null hypothesis, i.e. ${\vec{L}}^{v}$ is randomly oriented with respect to its discrete counterpart, predicts the null probability density distribution of θ to be sinθ (due to the infinitesimal solid angle dΩ=sinθdθdϕ), with the mean of 57.3° (1 rad) and the median of 60°. We use two-sided *t*-tests to see if the population averages of the mean and median of θ are different from these null values.

### 2.3. Effect Size

The diffusivity of the tissue can be quantified from $-log\left(\left|S^{v}(\overset{⇀}{q})\right|/S_{0}^{v}\right)$. In our case, using the DTI model, that would be:

[7] −log|Sv(q⇀)|S0v=τq⇀TDvq⇀−log1+(τL⇀vTDvq⇀)2≅τq⇀TDvq⇀−12(τL⇀vTDvq⇀)2=τq⇀TDv(I−12τL⇀vL⇀vTDv)q⇀.

The relative contribution of $\vec{L^{v}}$ to the above quantity, which we call the dimensionless *effect size*, is of the order of $1/2\tau {\left\|{\vec{L}}^{v}\right\|}_{2}^{2}\left\|D^{v}\right\|$. With the simplifying assumptions that PD remains relatively constant inside the voxel and *w* varies with a general linear trend, one can see ${\left\|{\vec{L}}^{v}\right\|}_{2}^{2}$ to be largely bounded by $4\left(1/s_{x}^{2}+1/s_{y}^{2}+1/s_{z}^{2}\right)$, where $s_{a}$ is the pixel size in the *a*-axis (these assumptions are used only here to derive this closed-form upper bound, and nowhere else). Therefore, for a dMRI of the brain white matter (WM) with $s_{x}=s_{y}=s_{z}=1.25\;mm$, τ=40ms, and $\left\|D^{v}\right\|=0.0007{\;mm}^{2}/s$, the effect size would be about 10^−4^ (enabling the approximation in Eq. [7]), which might be too small to detect. This is mainly due to the small particle displacement during the diffusion time, i.e. of the order of 10μm. The effect size can however be increased using a long τ. This prompted us to test our hypothesis additionally with the STE sequence(28,29) that can achieve a diffusion time of τ=1s (Section 2.4.2), while preserving the signal-to-noise ratio (SNR) by not increasing the effective TE (that would otherwise cause T_2_ signal loss).

It is also noteworthy that our hypothesized effect (Eq. [7]) is quadratic (rather than quartic) with respect to the q-vector, or linear (rather than quadratic) with respect to the b-value, meaning that it is not of a kurtosis (35) nature.

### 2.4. Population

We tested our algorithm on the following three datasets.

#### 2.4.1. Human Connectome Project (HCP) – Young Adult

We processed dMRI data of 617 subjects from the public WashU-UMN HCP Young Adult (27). The images had been acquired on a customized Siemens 3T scanner at the 1.25 mm isotropic resolution, with TR/TE = 5520/89.5 ms and the diffusion time of $\tau_{HCP}=40\;ms$, along 270 diffusion gradient directions with b-values ranging from 990 to 3010 s/mm^2^, in addition to 18 b=0 s/mm^2^ (*S*_0_) images. We used the brain (gray and white matter) mask computed from the T_1_ image via FreeSurfer (37), resampled it in the dMRI space, and refined it as described in Section 2.5.

#### 2.4.2. Acquired Stimulated-Echo (STE) dMRI

We used an STE dMRI (28,29) research sequence with the long diffusion time of $\tau_{STE}=1$ s to acquire the following images on a 3T scanner (MAGNETOM Skyra, Siemens Healthineers AG, Forchheim, Germany) with a 32-channel head coil, GRAPPA acceleration 2, b-value of 1000 s/mm^2^, and isotropic voxel size of 2 mm. A rough mask was used for analysis, as described in Section 2.5.

We scanned 10 healthy volunteers (7 females, age: 36 ± 14 years old), whose consent and data were collected as part of an existing project to develop novel MRI acquisition software, approved by the Institutional Review Board of Mass General Brigham. We used the receiver bandwidth 1658 Hz/px, TR of 26400 ms, effective TE of 31 ms, and image size of 104×104×25 voxels. We acquired images with 64 diffusion directions, as well as 20 repetitions of the b=0 image, resulting in a total acquisition time of 37.5 minutes.

In addition, we scanned an *ex vivo* sample of a brain hemisphere using the TR of 26200 ms, effective TE of 33 ms, receiver bandwidth 1698 Hz/px, and image size of 128×128×25 voxels. To increase the SNR, we acquired images with 256 diffusion directions, with 8 repetitions per direction, as well as 320 repetitions of the b=0 image, resulting in a total acquisition time of 17.3 hours. The repeated images were averaged before further analysis.

See Section 6 for the public availability of this long-diffusion-time STE dMRI dataset.

#### 2.4.3. Multi-TE (MTE) dMRI

We used the preprocessed dMRI images of the 3 subjects included in the public MTE dataset (30), acquired on a Siemens 3T Prisma scanner in 10 sessions (per subject) with 8 different TEs from 62 to 132 ms (with repeated measures at the shortest and longest TEs). The dataset included two b-values (700 and 2000 s/mm^2^) with 30 gradient directions for each b-value and 4 b=0 s/mm^2^ $\left(S_{0}\right)$ images. The isotropic voxel size was 2.5 mm, TR was 5800 ms, and the diffusion time was fixed at $\tau_{MTE}=20\;ms$. Preprocessing steps included denoising, corrections for B0 inhomogeneity, eddy current and motion correction, and aligning the images and b-vectors to the first TE session. We used the brain masks included in the dataset and refined them similarly to HCP, as described in Section 2.5.

### 2.5. Fiber Continuity: A Confounding Factor

Stemming from dMRI physics, our hypothesis is a relationship between diffusional information in the signal and spatial derivative of the image. However, characteristics of the fibrous tissue can additionally cause the fiber orientation to be confoundingly related to edges in the image. In particular, due to fiber continuity (38-40), fiber bundles vary smoothly along their own orientations, meaning that image edges would likely not be perpendicular to orientations with high diffusion. This means that the spatial gradient of the image in the fiber bundles is likely stronger along the lower- rather than higher-diffusion orientations.

In designing our experiments, we attempted to heuristically avoid the areas affected by fiber continuity by limiting our analysis to WM regions that are far from the bundle edges. For our *in vivo* (HCP, STE, and MTE) images, we only kept voxels with fractional anisotropy (FA) greater than 0.4, b=0 image intensity greater than 80, and mean ADC greater than 0.0002 mm^2^/s and smaller than 0.0008 mm^2^/s (HCP and MTE) or 0.0009 mm^2^/s (STE). For our *ex vivo* STE image, we used a rough mask including only regions with FA greater than 0.25, mean ADC smaller than 0.0003 mm^2^/s, and b=0 image intensity greater than 90.

Furthermore, for comparison, in addition to estimating the orientation of the spatial gradient of the image using our hypothesized relationship ${\vec{L}}^{v}$ in Eq. [5]), we also estimated it following the fiber continuity assumption as the orientation of the eigenvector corresponding to the smallest eigenvalue of the diffusion tensor, calling it ${\vec{F}}^{v}$.

## 3. Results

### 3.1. HCP Results

**Figure 1.**
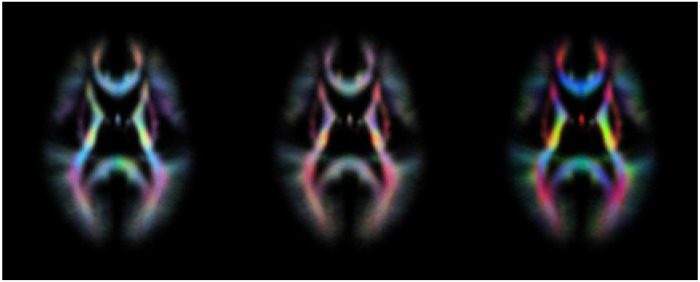
Axial view of the spatial gradient of the image estimated from the diffusion profile using the proposed (left) and fiber-continuity (right) approaches, and from the b=0 image using the finite-difference approach (middle). Colors show the strength of the gradient at each spatial coordinate after normalizing the gradient, taking its absolute value, and averaging across all HCP subjects (red = R/L, green = A/P, blue = S/I; not to be confused with color-coded FA).

We estimated the proposed ${\vec{L}}^{v}$, the fiber-continuity-derived ${\vec{F}}^{v}$, and their gold-standard discrete counterpart for each subject. Figure 1 shows the average of each of the three (normalized) vector fields across all the 617 subjects, color-coded with respect to the estimated orientation. One can see that the spatial-gradient orientations derived from both our (left) and fiber-continuity (right) approaches visually correspond to some extent to the discretely computed orientations (middle).

**Figure 2.**
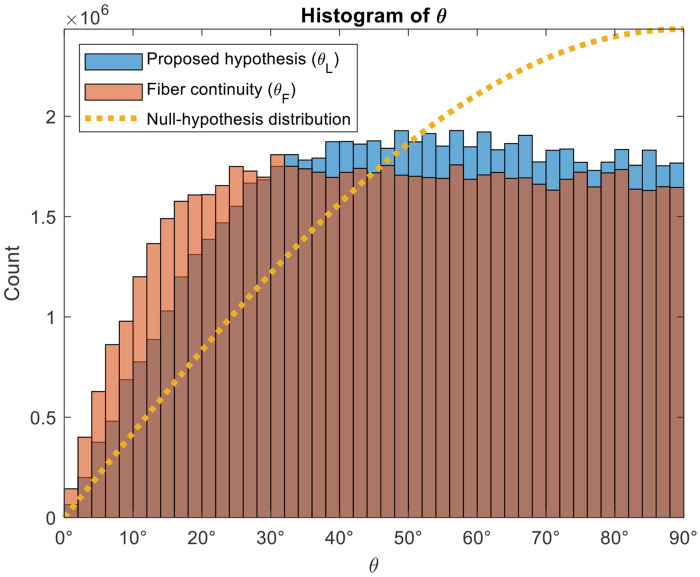
Histograms of the acute angles between spatial gradient of the image computed discretely from the b=0 image and the ones estimated from the diffusion profile using the proposed $\theta_{L}$, blue) and the fiber-continuity ($\theta_{F}$, red) approaches, across all WM voxels of all HCP subjects. The dotted orange line is the distribution of this angle under the null hypothesis (i.e., if the two orientations were not related).

The histogram of the acute angle $\theta_{L}$ between the estimated $\vec{L}$ and its discrete gold-standard counterpart across all WM voxels of all subjects is plotted in Figure 2. The distribution of $\theta_{L}$ (blue), which is visibly shifted to the left compared to the null hypothesis (dashed curve), has a mean/median of 51.3°/51.8°, i.e. smaller than chance (57.3°/60°). A similar histogram of $\theta_{F}$ between the fiber-continuity-derived ${\vec{F}}^{v}$ and the discrete counterpart is shown in red, which is further to the left, with its mean and median both being 48.6°.

**Figure 3.**
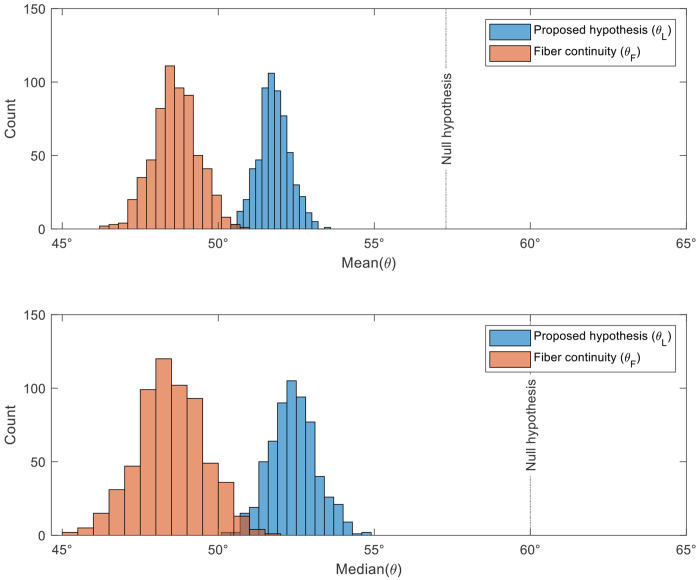
Histograms of the within-subject mean (top) and median (bottom) of the acute angles between spatial gradient of the image computed discretely from the b=0 image and the ones estimated from the diffusion profile using the proposed $\theta_{L}$, blue) and the fiber-continuity ($\theta_{F}$, red) approaches, across all HCP subjects. The dashed vertical lines indicate the values under the null hypothesis (i.e., if the two orientations were not related).

Next, we computed the mean/median of both $\theta_{L}$ and $\theta_{F}$ for each subject separately, the histograms of which across subjects are plotted in Figure 3. Two-tailed *t*-tests revealed these values to be significantly smaller than those predicted by chance (*p* = 0, within the double-precision limits).

**Figure 4.**
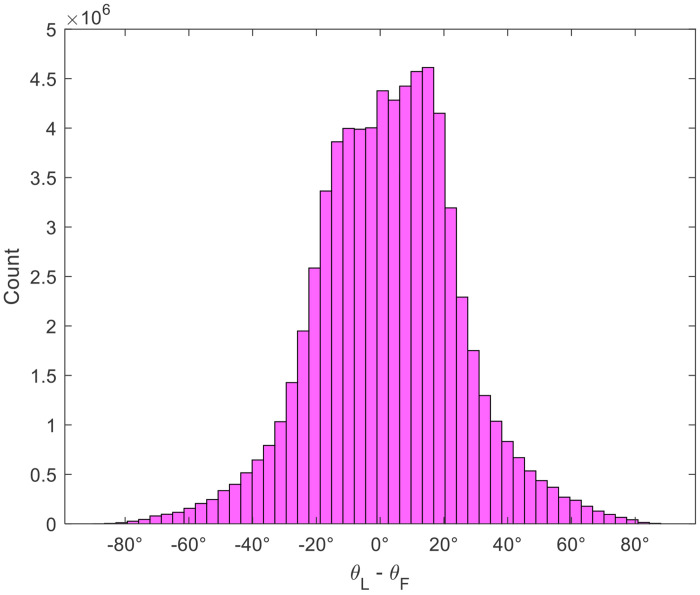
Histogram of $\theta_{L}-\theta_{F}$ across all WM voxels of all HCP subjects. 45% of the voxels are on the negative side $\left(\theta_{L}<\theta_{F}\right)$ of the distribution.

We then compared $\theta_{L}$ and $\theta_{F}$ voxel-wise. Even though $\theta_{L}$ was on average slightly larger than $\theta_{F}$ (see above), the histogram of $\theta_{L}-\theta_{F}$, plotted in Figure 4, revealed a considerable portion (45%) of voxels with $\theta_{L}<\theta_{F}$, implying *distinct* orientational information mined by the two approaches. The small effect size when comparing the two angles is further reflected in their pairwise Cohen’s *d* = 0.12, emphasizing that the two approaches to spatial gradient estimation are far from fully overlapping.

The diffusion tensor estimated from Eq. [5] on average had 0.973% (±0.003%) larger Frobenius norm, 1.161% (±0.004%) larger mean diffusivity, and 0.938% (±0.003%) smaller FA, compared to the tensor estimated the conventional way, i.e., by fixing $\vec{L^{v}}=0$ (errors are cross-subject standard error of the mean of the within-image mean value).

### 3.2. STE Results

**Figure 5.**
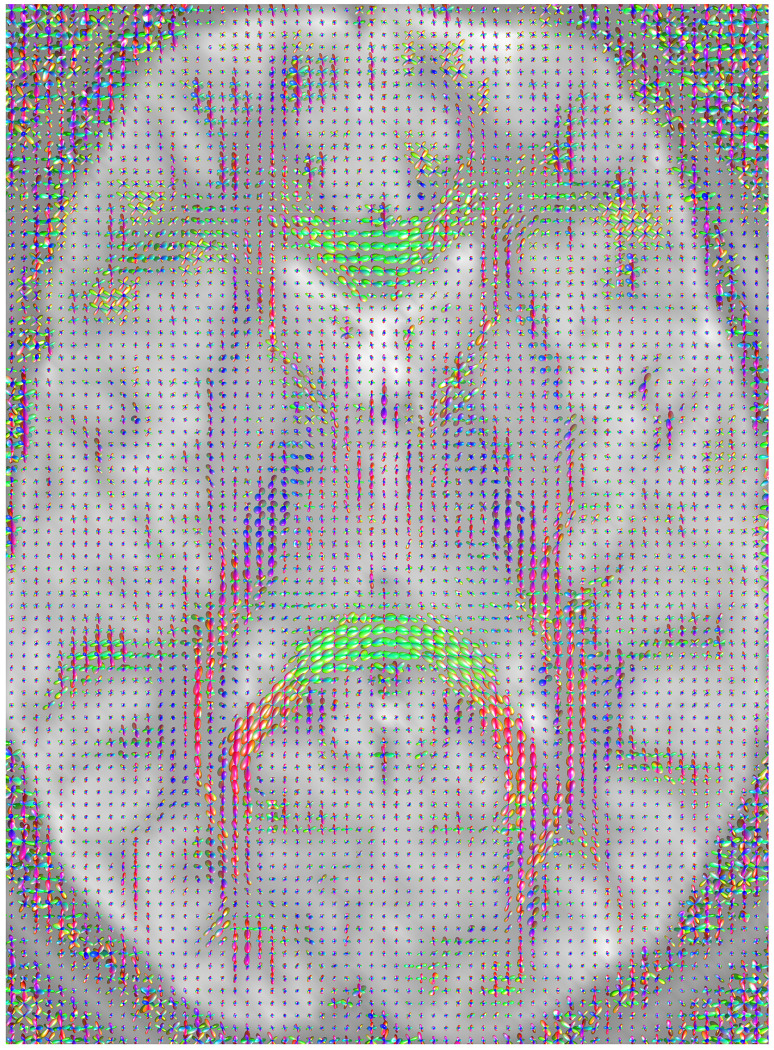
Axial view of the q-ball CSA-ODFs reconstructed from the STE data of a representative participant.

Figure 5 shows the q-ball orientation distribution functions in constant solid angle (CSA-ODFs) (41) reconstructed with a (real and symmetric) spherical harmonic order of 4 and visualized using our public MATLAB (The MathWorks Inc., Natick, MA, USA) toolbox (42) (see Section 6) for a representative subject in our *in vivo* STE dataset.

An analysis similar to Section 3.1 for our *in vivo* STE images revealed the mean/median of $\theta_{L}$ and $\theta_{F}$ to be 53.0°/54.5° and 44.7°/42.7°, respectively, which are again smaller than chance (57.3°/60°). Within-subject mean/median of both $\theta_{L}$ and $\theta_{F}$ were significantly smaller than those predicted by chance (cross-subject *t*-test *p* < 10^−10^). Comparing $\theta_{L}$ and $\theta_{F}$ voxel-wise revealed 39% of voxels with $\theta_{L}<\theta_{F}$.

**Figure 6.**
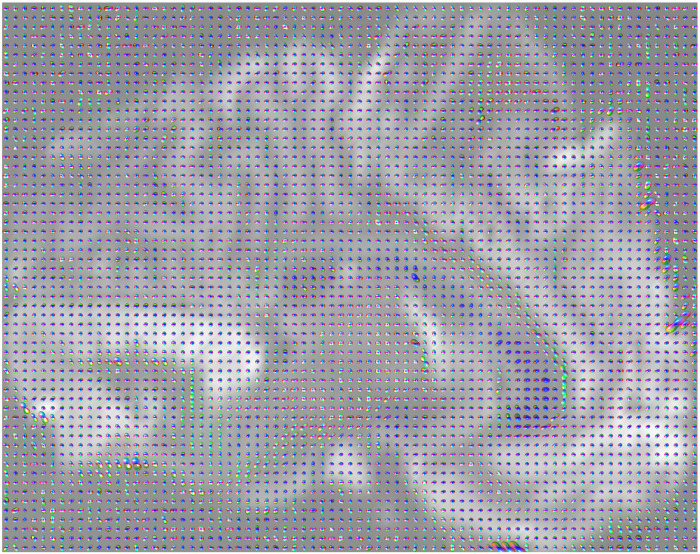
Sagittal view of the DTI CSA-ODFs reconstructed from the *ex vivo* STE scan.

Regarding our *ex vivo* STE image, the CSA-ODFs reconstructed from the diffusion tensors are depicted in Figure 6. The mean/median of $\theta_{L}$ and $\theta_{F}$ for this image were 56.5°/58.7° and 53.5°/55.2°, respectively, which are smaller than chance (but not by as much as the *in vivo* results), with 46% of voxels showing $\theta_{L}<\theta_{F}$.

### 3.3. MTE Results

An analysis similar to Section 3.1 over all subjects and acquisitions revealed the mean/median of $\theta_{L}$ and $\theta_{F}$ to be 53.6°/55.0° and 47.7°/47.2°, respectively, i.e., smaller than chance. Within-image mean/median of both $\theta_{L}$ and $\theta_{F}$ were significantly smaller than those predicted by chance (cross-image *t*-test *p* < 10^−29^). Comparing $\theta_{L}$ and $\theta_{F}$ voxel-wise revealed 41% of voxels with $\theta_{L}<\theta_{F}$.

**Figure 7.**
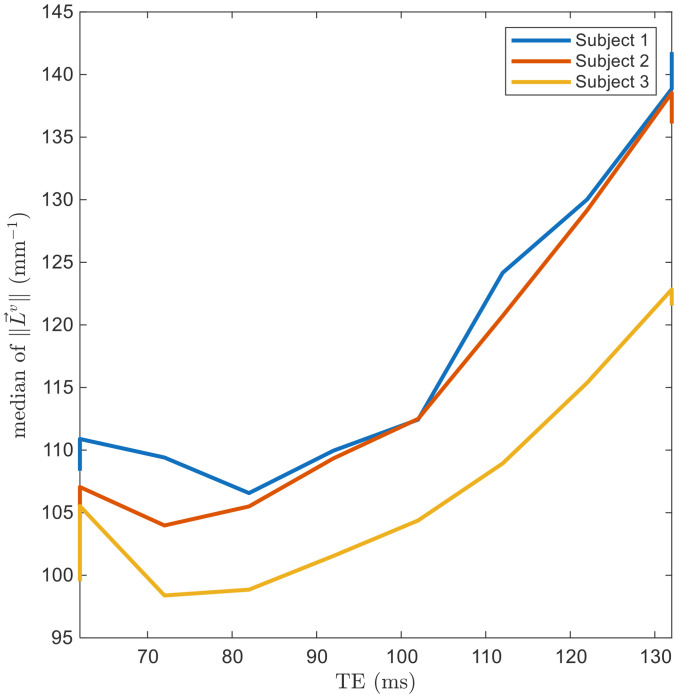
Within-image median of the magnitude of the estimated ${\vec{L}}^{v}$ in the MTE dataset for each TE.

Given the long TR of the data, the RT weighting would chiefly reflect the T_2_ weighting, $w(\vec{x})≅exp\left(-TE/T_{2}(\vec{x})\right)$, resulting in ${\vec{L}}^{v}={\left\langle \nabla_{x}w\right\rangle }_{\rho }^{v}/\langle w\rangle_{\rho }^{v}=-TE{\left\langle \nabla_{x}\left(1/T_{2}(\vec{x})\right)exp\left(-TE/T_{2}(\vec{x})\right)\right\rangle }_{\rho }^{v}/{\left\langle exp\left(-TE/T_{2}(\vec{x})\right)\right\rangle }_{\rho }^{v}$, with the magnitude ${\left\|\vec{L^{v}}\right\|}_{2}\propto TE$ (roughly). We indeed observed that the within-image median of our estimated ${\left\|{\vec{L}}^{v}\right\|}_{2}$ generally increased with *TE* (after an initial dip), as shown in Figure 7. A linear mixed-effects analysis, relating median ${\left\|\vec{L^{v}}\right\|}_{2}$ to *TE*, with the subject as the random effect, revealed a significant positive relationship (*p* = 10^−6^).

Next, we applied MATLAB’s exponential curve fitting tool to fit all the images of each subject to $S_{0}^{v}=\rho^{v}exp\left(-TE/T_{2}^{v}\right)$ to estimate $T_{2}^{v}$ and the PD, $\rho^{v}$, for each voxel *v*, with positivity constraints for both variables. Unfortunately, the resulting $T_{2}^{v}$ (and $w^{v}≅exp\left(-TE/T_{2}^{v}\right)$) maps were not of good quality. To increase the SNR (especially since we had fewer b=0 images for each dMRI scan than in the other datasets), we replaced *S*_0_ with the average of the entire set of images (b=0 and diffusion-weighted) to be used as the discrete gold standard for this dataset. While this improved the overall results, the estimated $w^{v}$ still had slice-wise intensity inhomogeneities that compromised the accuracy of our finite-difference estimate of the gradient of $w^{v}$, i.e. $\partial_{fd}w^{v}$, at least in the slice-select direction. We computed the within-image median of $\theta_{L}$ for each subject and TE, but this time using the estimated $w^{v}$ or $\rho^{v}$ as the gold standard. For the first subject, using either $w^{v}$ or $\rho^{v}$ did not improve (decrease) $\theta_{L}$ over the unseparated (original) image. For the second subject, using $w^{v}$ and $\rho^{v}$ resulted in smaller $\theta_{L}$ than the unseparated image did by 0.85° and 0.84°, respectively, while for the third subject, it did so by 0.32° and 0.79°, respectively.

## 4. Discussion

The spatial gradient of the RT weighting stems from local variations in the molecular composition of the biological tissue (water, fat, macromolecule, and paramagnetic substance contents), which are generally due to the spatial variations in the biophysical properties of the healthy tissue, but can also be a result of heterogeneities caused by pathology (edema, tumors, hemorrhage, demyelination, etc.) (15,43–45). We have introduced a model that takes advantage of diffusional information in the dMRI signal to infer new spatial information, thereby deriving a new relationship between the diffusion profile and the spatial gradient of tissue RT weighting. We evaluated our model on 617 brain dMRI images from the public HCP database, on 10 *in vivo* and 1 *ex vivo* brain STE images that we acquired at our Center, and on 30 brain dMRI images (with various TEs) of 3 subjects from the MTE dataset. We observed the effect of our hypothesized relationship in all our results; namely, the spatial-gradient orientation estimated from the diffusion profile using our relationship was statistically significantly closer to the gold-standard orientation (approximated through finite difference) than predicted by chance.

While the relationship between diffusional and RT properties of the tissue has been discussed (12–15) and measured (16–21,46) in the literature, our proposed framework is – to the best of our knowledge – the first to enable the estimation of the within-voxel spatial gradient of the RT weighting from the dMRI signal. In the work most relevant to ours (15), the authors present a detailed mathematical framework that predicts how the measured dMRI signal is affected by the diffusion time, the intrinsic variations of the Larmor frequency, and the intrinsic variations of the transverse relaxation rate, $R_{2}(\vec{x})=1/T_{2}(\vec{x})$. For the latter case, they derive a general integral formula containing the Fourier transform of the autocorrelation function of $R_{2}(\vec{x})$. Our mathematical approach, however, is distinct from that of (15); in particular, we calculate the effects of variations of the entire RT weighting, $w(\vec{x})$, thereby naturally accounting for not only the transverse $\left(T_{2}(\vec{x})\right)$ but – as opposed to (15) – also the longitudinal $\left(T_{1}(\vec{x})\right)$ RTs (although the effects of the latter are small in dMRI). Furthermore, we derive a closed-form formula (Eq. [5]) that enables the direct estimation of the spatial gradient of $w(\vec{x})$, whereas, to the best of our knowledge, the derivations in (15) do not provide a straightforward means to estimate the spatial gradient (of $w(\vec{x})$ or $R_{2}(\vec{x})$).

The decision to test our hypothesis additionally on the STE images was motivated by the theoretical prediction that the spatial image gradient affects the signal more strongly at longer diffusion times (Section 2.3). Our STE protocol with $\tau_{STE}=1\;s$ indeed provided us with a diffusion time 25 times longer than that of HCP ($\tau_{HCP}=40\;ms$), although, with its larger voxel size, the predicted effect size would have been only 9.8 times larger (see Section 2.3). Nevertheless, the effect observed in the STE images was not as strong as that in the standard dMRI images of HCP, possibly due to other differences between the two datasets (see Section 4.1).

A confounding factor in validating our hypothesis was the concept of fiber continuity (38–40), a characteristic of the fibrous tissue that implies smooth variation of a fiber bundle along its orientation, hence smaller image gradient along high-diffusion orientations (see Section 2.5). To distinguish the relationship derived from our dMRI-physics hypothesis from that due to fiber continuity, we also estimated the spatial gradient of the image from the latter relationship, which produced, on average, more accurate spatial-gradient orientations (when compared to the gold standard) than our hypothesis did. Nevertheless, we observed that in a substantial portion of the voxels (45% in HCP, 39% in *in vivo* STE, 46% in *ex vivo* STE, and 41% in MTE), our hypothesized relationship still led to more accurate orientation estimation than fiber continuity did. This implies that, regardless of the fiber continuity effect being stronger than our hypothesized effect, the latter is not simply a weaker version of the former. In other words, our observations rule out the (full) overlap of the two effects, which can be clearly observed in the broad distribution of the difference between the acute angles estimated from our and fiber-continuity approaches (Figure 4).

The diffusion tensor estimated from our model showed slightly higher diffusivity compared to the conventional tensor. This is expected, since the square root in Eq. [5] is greater than 1, and a higher diffusivity in the exponential is needed to compensate for that. Our tensors were also slightly more isotropic, which could be because some of the anisotropy in the signal was covered by the new factor related to the spatial gradient.

Potential future directions would be to investigate whether the spatial information hidden within the diffusion profile could: 1) help to improve the characterization of fine WM pathways and cortical microstructure, especially given that the spatial gradient for a voxel is estimated at the microscale (rather than the mesoscale as the finite-element approach does using neighboring voxels), 2) be used as a dMRI biomarker for disease, and 3) be exploited as supplemental input to improve dMRI super-resolution algorithms (47–50), given that our microscopic and the finite-element mesoscopic approaches can jointly and complementarily inform the super-resolution method about the image spatial gradient. The latter is especially important as higher dMRI spatial resolution reduces the unwanted mixture of tissues in a voxel (partial volume effect), enhancing the precision and reliability of diffusion modeling and tractography (7,51–53).

Our STE dMRI dataset, which is publicly available (see Section 6), is unique in that it has been acquired with an unusually long diffusion time of $\tau_{STE}=1\;s$. This dataset includes *in vivo* brain images of 10 healthy volunteers and 1 *ex vivo* brain image. While we collected these data specifically to test the hypothesis proposed here, we hope that this dataset will be useful for the research community to study the diffusion properties of the neural tissue at very long diffusion times (54), e.g. the restricted diffusion (55), and test other hypotheses. Public STE datasets by other groups, e.g. of the human heart (56) or the mouse spinal cord (57), can further facilitate such efforts.

### 4.1. Limitations

Although we showed that the effects of the proposed model did not fully overlap with those of fiber continuity, our significant results may still be due to a mix of the two effects. Further evaluation, e.g. on physical phantoms constructed while minimizing the fiber continuity effect, can be helpful to conclusively validate our hypothesis.

In our evaluation, where the discretely computed image gradient was used for comparison, we made an approximation by ignoring the variations in the PD image in Eq. [6]. Nonetheless, since we used only the orientation (but not the magnitude) of the spatial gradient for validation, and given that the gradients of the PD and b=0 images are both related to the same underlying tissue and thus oriented similarly to each other, the aforementioned approximation is not expected to have substantially affected the accuracy of our validation. Another consideration about our evaluation is that the gold-standard gradient was computed as the *central* finite difference, which, by using multiple voxels, introduces some blurring that is not expected in our diffusion-based model, hence a discrepancy in the comparison. While our results show a statistically significant relation between the gradients estimated through our and the finite-element methods, the effect size was not large *per se* (as predicted in Section 2.3). Our approach in fact quantifies the spatial gradient using within-voxel microscopic information hidden in the diffusion signal, whereas the finite-element approach does so using mesoscopic information from neighboring voxels. Therefore, the two measures are of different natures and affected by distinct sources of error.

To test our hypothesis, we applied the DTI model due to its simplicity and widespread use. This model’s ability to represent the diffusion signal, however, is limited in regions with fiber crossing and where the diffusion profile deviates from Gaussian (especially at high b-values). We attempted to alleviate these by limiting our analysis to regions with FA values above a certain threshold. Nevertheless, extending Eq. [5] to higher models, such as diffusional kurtosis imaging (35), can further help to overcome the limitations of DTI. Systematic effects such as B1 field inhomogeneity, point spread function, and Gibbs ringing may also have affected the gradient estimated via both our and the finite-element approaches, which should be considered when interpreting our results.

Our hypothesized effect was observed to be weaker for the STE compared to the HCP data, possibly because of the larger voxels of the STE images ($s_{x}=s_{y}=s_{z}=2\;mm$, compared to $s_{x}=s_{y}=s_{z}=1.25$ mm of the HCP; see Section 2.3), fewer diffusion images, lower SNR (two-time reduction in signal strength) inherent to STE (29), inadequacy of the DTI (Gaussian) model at long τ (58), and less specific WM mask.

## 5. Conclusions

We extended the standard dMRI reconstruction model to account for within-voxel variation of the RT properties of the tissue, and derived a closed-form relationship in the case of DTI. The new mathematical model enables the estimation of the spatial image gradient from the diffusion profile. We evaluated our model on public HCP data, as well as a unique in-house STE dataset that we acquired with a very long diffusion time (and provide freely to the public). Our experimental results support the validity of our hypothesis with statistical significance. Future work consists of leveraging the estimated spatial gradient to learn about tissue microstructure, discover biomarkers, and increase the spatial resolution of dMRI.

## 6. Data and Code Availability

We have curated, de-identified, and de-faced our acquired STE dataset described in Section 2.4.2, and will make it publicly available (as soon as our data use agreement is finalized by our institution), providing the link to the dataset in the final version of the manuscript.

Our MATLAB code for the proposed spatial-gradient estimation is included as the estimateSpatialGradient function in our public CSA-ODF and Hough-Tractography toolbox (42) (www.nitrc.org/projects/csaodf-hough). Diffusion and STE fiber orientation reconstruction was performed using the same toolbox.

The public WashU-UMN Human Connectome Project (HCP) Young Adult (27) database is available at: https://www.humanconnectome.org/study/hcp-young-adult/data-releases

The public Multi-TE (MTE) dataset (30) is available at: https://doi.org/10.57760/sciencedb.o00133.00038
